# Expression and functional analysis of the transcription factor-encoding Gene *CsERF004* in cucumber during *Pseudoperonospora cubensis* and *Corynespora cassiicola* infection

**DOI:** 10.1186/s12870-017-1049-8

**Published:** 2017-06-05

**Authors:** Dong Liu, Ming Xin, Xiuyan Zhou, Chunhua Wang, Yanju Zhang, Zhiwei Qin

**Affiliations:** 1 College of Horticulture and Landscape Architecture, Key Laboratory of Biology and Genetic Improvement of Horticultural Crops (Northeast Region), Northeast Agricultural University, Harbin, 150030 China; 2 College of Agriculture, Northeast Agricultural University, Harbin, 150030 China; 3 Heilongjiang Bayi Agricultural University, Daqing, 163319 China

**Keywords:** Cucumber downy mildew, Cucumber target spot, *CsERF004*, Disease resistance

## Abstract

**Background:**

Cucumber downy mildew, caused by *P. cubensis*, is an important leaf disease that can severely affect cucumber production. In recent years, cucumber target spot, caused by *C. cassiicola*, has been reported in both Asia and Europe and is now considered as a major disease disrupting cucumber production. Single-disease-resistant cucumber varieties have been unable to satisfy production needs.

To explore the molecular mechanisms of cucumber resistance to these two diseases, cucumber cultivars D9320 (resistant to downy mildew and target spot) and D0401 (susceptible to downy mildew and target spot) were used as experimental materials in this study. We used transcriptome sequencing technology to identify genes related to disease resistance and verified using transgenic technology.

**Results:**

We screened out the cucumber resistance-related gene *CsERF004* using transcriptome sequencing technology. Induction by pathogens, salicylic acid (SA), and ethylene (ET) resulted in the up-regulation of *CsERF004*. Three treatments, namely, inoculation with *C. cassiicola* alone, inoculation with *P. cubensis* alone, and simultaneous inoculation with both pathogens, all resulted in the significant and sustained up-regulation of *CsERF004* in the resistant cultivar D9320, during the early stage of infection. In the susceptible cultivar D0401, *CsERF004* expression was also significantly up-regulated at the later stage of infection but to a lesser extent and for a shorter duration than in the resistant cultivar D9320. The *CsERF004* gene encodes a protein localizes to the nucleus. The over-expression of *CsERF004* in the susceptible cultivar D0401 resulted in the significant up-regulation of the *CsPR1* and *CsPR4* genes and increased the levels of SA and ET, which enhanced the resistance of cucumber to downy mildew and target spot.

**Conclusions:**

Analyses of the *CsERF004* expression pattern in disease-resistant and susceptible cucumber cultivars and transgenic validation indicate that *CsERF004* confers resistance to *P. cubensis* and *C. cassiicola*. The findings of this study can help to better understanding of mechanisms of response to pathogens and in establishment the genetic basis for the development of cucumber broad-spectrum resistant cultivars.

**Electronic supplementary material:**

The online version of this article (doi:10.1186/s12870-017-1049-8) contains supplementary material, which is available to authorized users.

## Background

Cucumber (*Cucumis sativus* L.) is a staple vegetable that produces tender edible fruits. In production, cucumber is susceptible to a variety of infections that severely affect its yield and quality. Two major diseases that affect cucumber production are cucumber downy mildew caused by *Pseudoperonospora cubensis* (Berk. & M.A.Curtis, Rostovzev) [1] and cucumber target spot caused by *Corynespora cassiicola* (Berk & Curt, Wei) [2]. Disease epidemics involving these pathogens generally spread quickly, disrupting cucumber production. Downy mildew and target spot, reduce cucumber yields by 50% [3] and 60–70% [4], respectively. The production and quality of cucumber have been affected resulting in economic losses. There is some controversy about genetic control of resistance to downy mildew and target spot. Some studies reported that resistance to downy mildew is controlled by multiple genes [5–9], while others believe it to result from a single recessive gene [10–13]. Resistance to target spot is generally believed to be controlled by a single gene. However, whether this gene is dominant or recessive has been discussed. Several studies have shown that resistance is controlled by a single dominant gene [14], while others indicate that it is controlled by a single recessive gene [15–17]. The discrepancies in the above results may be related to the use of different experimental materials. The use of single-disease-resistant cucumber varieties has already proven insufficient to prevent losses in cucumber production due to these diseases. Therefore, the study of broad-spectrum disease resistance mechanisms can be important to establish the genetic basis for the development of multi-disease-resistant cucumber varieties.

Unlike mammals, plants are not equipped with mobile cells and do not possess an adaptive immune system. However, during co-evolution with pathogens, plants developed an innate immune system [18] consisting of two levels. The first level involves pathogen-associated molecular pattern-triggered immunity (PTI), which can prevent some pathogens from entering the cell via oxygen bursts and callose deposition [19–21]. The second level consists of the specific recognition of resistance proteins and avirulence proteins, triggering the hypersensitive response and inhibiting the growth of pathogenic bacteria via programmed cell death in a process known as effector-triggered immunity (ETI) [22]. Transcription factors can activate or inhibit the expression of genes related to disease resistance; thus, they play an important role in disease resistance in plants [23]. The AP2/ERF-like transcription factors compose a large family of proteins that is divided into five subfamilies based on the number of AP2/ERF domains; they include AP2, RAV, DREB, ERF, and others [24]. The ERFs are a major subfamily of the AP2/ERF transcription factor family and occur widely in plants. ERF-subfamily transcription factors have been isolated from *Arabidopsis thaliana* [25], tobacco [26], soybean [27], rice [28], maize [29], and tomato [30]. ERFs can regulate the expression of the *PR* genes in combination with the GCC-box and DRE/CRT cis-acting elements in the promoter region of the gene [31, 32]. *PR* genes play an important role in plant resistance to various infections [33]. Expression of *PR-1a*, *PR4*, *PR5*, and *PR10* genes has been associated with resistance to pathogen infection and has been shown to improve plant disease resistance [34–37]. ERF transcription factors are involved in a variety of plant hormone signaling pathways [38, 39], and are the connecting factors of signal cross-linking pathway under stress, thus playing an important role in plant growth and development as well as in resistance to biotic and abiotic stresses [40–44]. Over-expression of *ERF* genes in rice [44] and soybean [23] has been shown to increase plant disease resistance.

To date, a total of 103 genes that encode complete AP2/ERF domains have been identified in the cucumber genome [45]. However, our understanding of the function of the cucumber *ERF* gene subfamily is limited because only a single gene, *CsERF* (Csa7G448110), has been reported to regulate the expression of the bitter gene *Bi* [46]. Furthermore, ERF has not been reported that regulates to cucumber disease resistance. In the present study, we cloned the *CsERF004* gene, analysed its expression patterns after inoculation with only *C. cassiicola*, only *P. cubensis* or both pathogens, and explored its role in disease resistance using transgenic validation in cucumber, thereby laying the foundation for cultivating multi-resistant cucumber varieties.

## Results

### Screening of disease-related genes

**Fig. 1:**
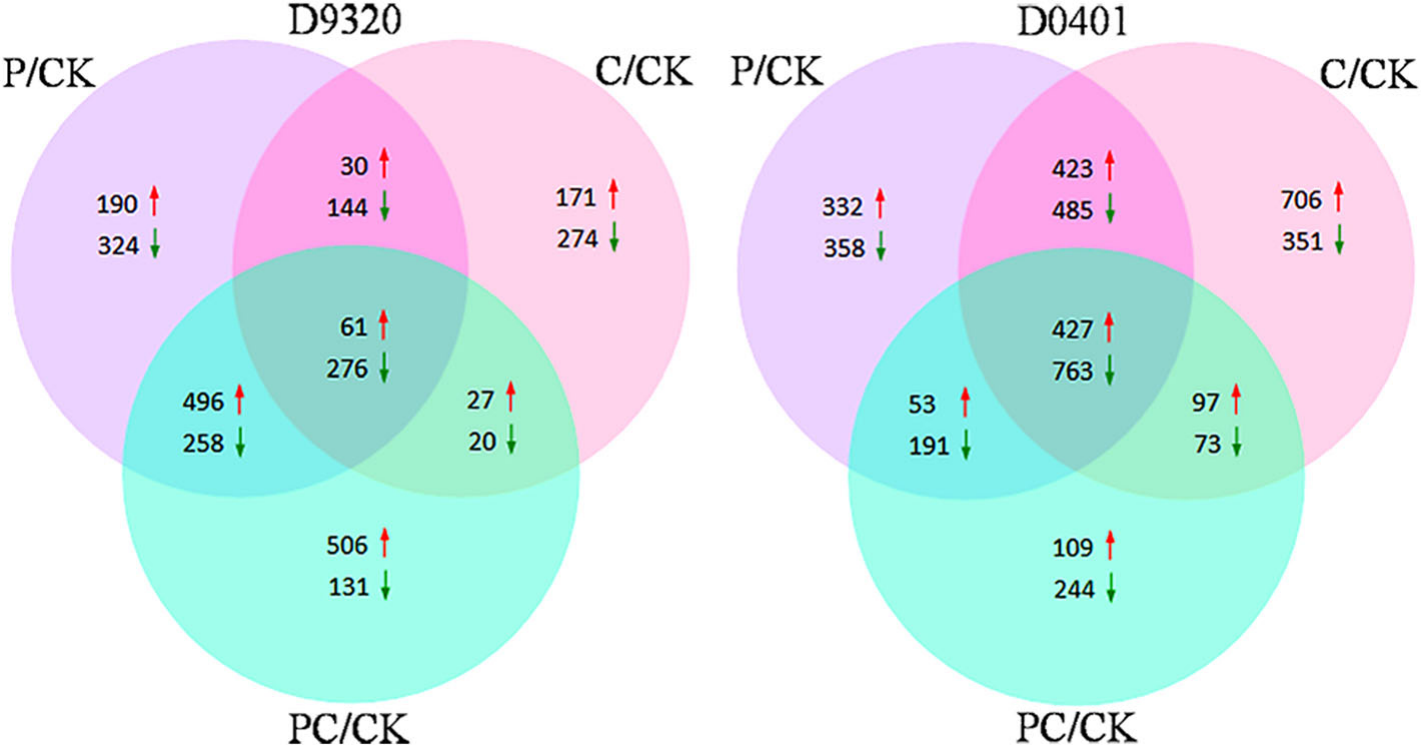
Differentially expressed genes venn diagrams of cucumber cultivars D9320 and D0401 under pathogen stress. D9320: resistant cultivar. D0401: susceptible cultivar. P/CK represents a set of differentially expressed genes of comparison between the *P. cubensis*-infected sample and the control sample at 48 h. C/CK represents a set of differentially expressed genes of comparison between the *C. cassiicola*-infected sample and the control sample at 48 h. PC/CK represents a set of differentially expressed genes of comparison between the two pathogens-infected sample and the control sample at 48 h. Sterilized water was used as a control. *Red arrows* indicate up-regulated genes in infected samples, and *green arrows* represent down-regulated genes in infected samples

The raw data were uploaded to the National Center for Biotechnology Information (NCBI) (GenBank accession no.SRX2468535). Transcriptome sequencing after the three treatments identified 61 up-regulated and 276 down-regulated genes in the resistant cultivar D9320 and 427 up-regulated and 763 down-regulated genes in the susceptible cultivar D0401 (Fig. 1). A total of 61 genes were up-regulated in the D9320 genotype under all three inoculation conditions; 16 of them were also up-regulated in the D0401 genotype and were not analysed further. The remaining genes that were up-regulated in the D9320 genotype were either down-regulated or not differentially expressed in the D0401 genotype. According to annotations in the genome database, 10 of these genes may be associated with disease resistance (Table 1). The over-expression of ERF transcription factors can enhance resistance to two or three diseases [41, 44]. The purpose of our study was to explore the mechanism of resistance to two diseases in cucumber. Therefore, the *CsERF004* gene (Csa7M432080.1) was selected to study the mechanism of resistance to two diseases in cucumber.

**Table 1:** Expression levels of resistance-related genes in cucumber cultivars D9320 and D0401 after different treatments

Gene ID	D9320			D0401		
	log_2_(Ratio) (C/CK)	log_2_(Ratio) (P/CK)	log_2_(Ratio) (PC/CK)	log_2_(Ratio) (C/CK)	log_2_(Ratio) (P/CK)	log_2_(Ratio) (PC/CK)
Csa2M350210.1	1.86	1.79	2.15	−4.48	−3.25	−2.22
Csa2M361700.1	1.90	2.00	1.53	−1.63	−1.56	—
Csa3M002970.1	1.10	1.16	1.08	−1.88	−3.77	—
Csa3M791530.1	1.76	2.20	2.16	—	—	—
Csa3M826660.1	2.58	1.89	1.77	−3.27	−2.12	−1.18
Csa5M152920.1	2.66	3.64	3.46	−2.61	−1.44	—
Csa5M466350.1	1.61	1.17	1.07	—	—	—
Csa6M006890.1	1.04	1.58	1.28	—	—	—
Csa6M496430.1	1.05	1.28	1.51	—	—	—
Csa7M432080.1	1.38	3.53	2.74	−1.57	—	—

C/CK represents differentially expressed genes of comparison between the *C. cassiicola*-infected sample and the control sample at 48 h. P/CK represents differentially expressed genes of comparison between the *P. cubensis*-infected sample and the control sample at 48 h. PC/CK represents differentially expressed genes of comparison between the two pathogens-infected sample and the control sample at 48 h. Spraying sterilized water was used as a control

Differentially expressed genes were identified based on a *p* value ≤0.01 and | log2 ratio ≥ 1 |. “—” indicates a non-differentially expressed gene. Non-differentially expressed genes did not meet the screening conditions

### Gene cloning and bioinformatics analysis

**Fig. 2:**
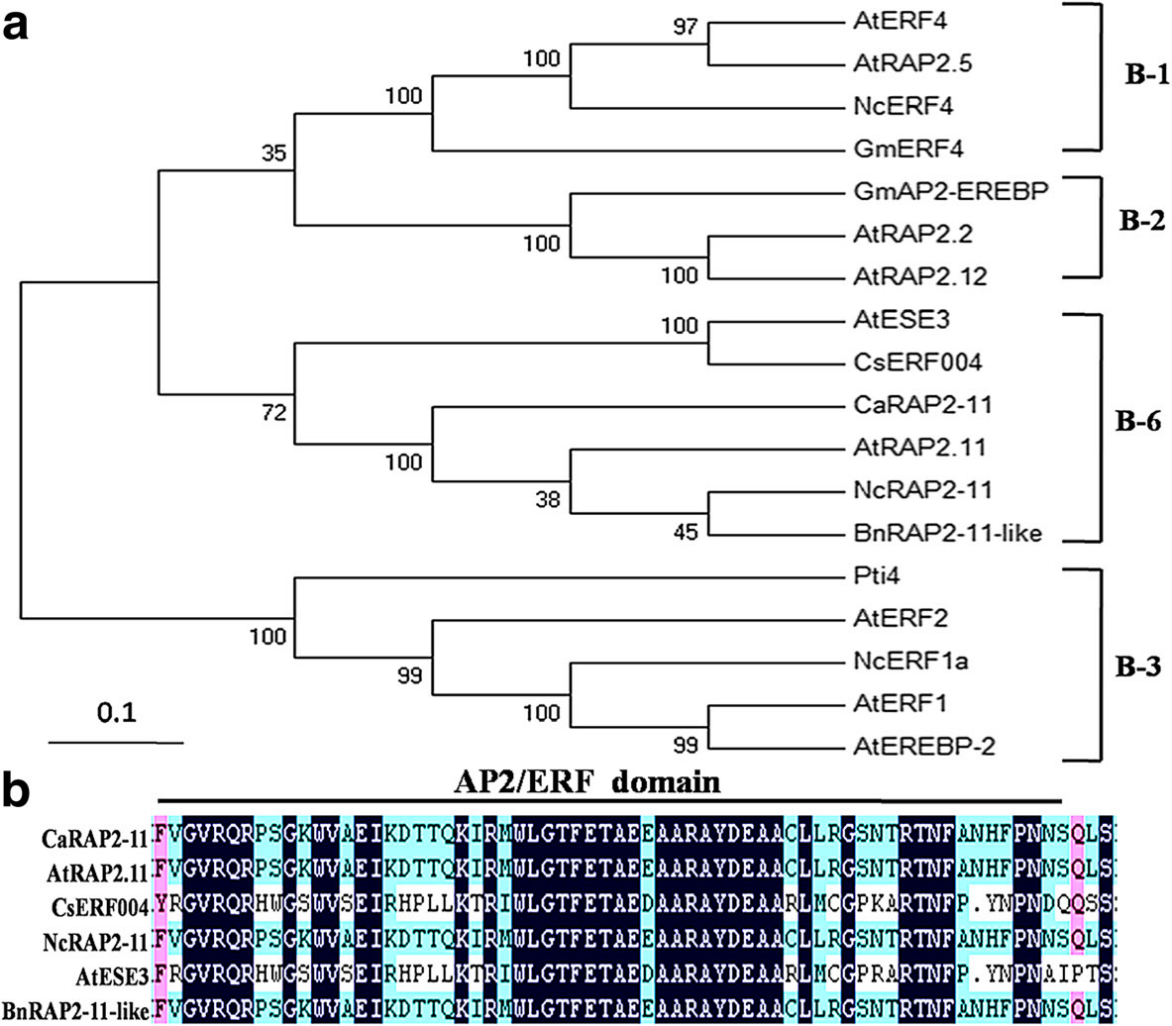
Phylogenetic analysis and sequence alignment of CsERF004*.* **a** Phylogenetic analysis of CsERF004 with ERF proteins; the phylogenetic tree was constructed using the amino acid sequences of the AP2/ERF domains in each ERF protein. The GenBank accession numbers are as follows: AtRAP2.11 (AAC49777.1), NcRAP2–11 (JAU24088.1), CaRAP2–11 (XP_010493057.1), BnRAP2–11-like (XP_013667343.1), AtESE3 (NP_197901.1), AtERF1 (NP_567530.4), NcERF1a (JAU16327.1), AtEREBP-2 (CAB45963.1), AtERF2 (NP_199533.1), Pti4 (NP_001275437.1), AtRAP2.2 (NP_850582.1), AtRAP2.12 (NP_175794.1), GmAP2-EREBP (NP_001238300.1), GmERF4 (NP_001238595.1), AtERF4 (NP_188139.1), AtRAP2.5 (AAC49771.1), and NcERF4 (JAU43921.1). **b** The conserved AP2/ERF domain of the B-6 group proteins. The conserved AP2/ERF domain is marked with a *black horizontal solid line*

The *CsERF004* gene was cloned from the resistant cucumber cultivar D9320 by PCR using cDNA extracted from cucumber leaves as a template; its CDS was 591 bp in length and encoded 196 amino acids. The protein encoded by this gene has one AP2/ERF domain, and belongs to the ERF transcription factor B-6 family. (Fig. 2).

### Analysis of the *CsERF004* gene expression pattern under pathogen stress

After inoculation with either *P. cubensis* or *C. cassiicola* as well as after inoculation of both pathogens, the expression of the *CsERF004* gene was differed between the resistant cultivar D9320 and the susceptible cultivar D0401.

**Fig. 3:**
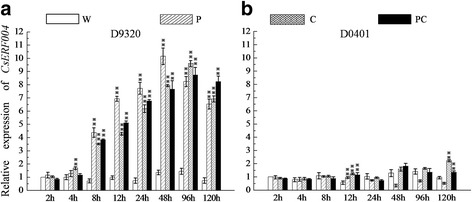
Relative expression levels of the *CsERF004* gene in two cucumber varieties and after three treatments. **a** and **b** represent the relative expression levels of *CsERF004* in the resistant cultivar D9320 and susceptible cultivar D0401, respectively. W: water control. P: inoculation of *P. cubensis*. C: inoculation of *C. cassiicola*. PC: inoculation of both pathogens. There were three biological replicates per treatment and three technical replicates per sample. Student’s t-test was used for comparison between two samples. “**” indicates that the difference between the corresponding column value and the adjacent control column value is significant

After inoculation with *P. cubensis*, the expression level of the *CsERF004* gene in the D9320 genotype was significantly increased at 8 h, and reached a peak at 48 h (approximately 10-fold), and then decreased slightly, but yet remained higher than that of the control, (Fig. 3a). The expression level of the *CsERF004* gene in the D0401 genotype was only significantly increased at 12 h (Fig. 3b).

After inoculation with *C. cassiicola*, the expression level of *CsERF004* gene in the D9320 genotype was significantly increased at 4 h, and reached a peak at 96 h (approximately 9.5-fold), and then decreased slightly, but yet remained higher than that of the control (Fig. 3a). The expression level of *CsERF004* gene in the D0401 genotype was significantly increased at 12 h (1–1.5-fold) and 120 h (approximately 2.5-fold) (Fig. 3b).

After inoculation with both pathogens, the expression level of *CsERF004* gene in the D9320 genotype was significantly increased at 8 h, and reached a peak at 96 h (approximately 8.5-fold), and then decreased slightly, but yet remained higher than that of the control (Fig. 3a). The expression level of *CsERF004* gene in the D0401 genotype was significantly increased at 12 h (1–1.5-fold) and 120 h (approximately 1.5-fold) (Fig. 3b).

The above results show that the expression of *CsERF004* gene was significantly up-regulated in the resistant cultivar D9320 during the early stage of infection, and this expression was high and long-lasting. In the susceptible cultivar D0401, the expression of *CsERF004* gene was also significantly up-regulated at the later stage of infection, although this induction was comparatively lower and shorter in duration than that in D9320.

Gene expression patterns are often associated with gene function [47]. Differences in the expression of the *CsERF004* gene in response to *P. cubensis* and *C. cassiicola* were observed between the resistant cultivar D9320 and the susceptible cultivar D0401, indicating that the *CsERF004* gene might be closely associated with resistance to downy mildew and target spot.

### Analysis of the *CsERF004* gene expression pattern in response to hormone induction

**Fig. 4:**
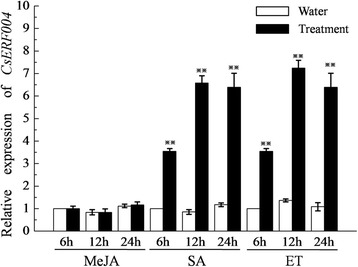
Relative expression levels of *CsERF004* induced by different hormones in the resistant cultivar D9320. There were three biological replicates per treatment and three technical replicates per sample. Sterilized water was used as a control. Student’s t-test was used to compare two samples. “**” indicates that the difference between the corresponding column value and the adjacent control column value is significant

Plant resistance to diseases involves hormone signal transduction pathways. ERF transcription factors are involved in a variety of hormone responses [38, 39]. In the present study, the resistant cucumber cultivar D9320 was treated with methyl jasmonate (MeJA), salicylic acid (SA) and ethylene(ET), respectively. After MeJA treatment, *CsERF004* expression did not significantly change (Fig. 4). After SA treatment, *CsERF004* was significantly up-regulated and reached its peak expression at 12 h, with an expression level 6.5–7-fold higher than that in the control (Fig. 4). After ET treatment, the expression of *CsERF004* also was significantly up-regulated and peaked at 12 h, with an expression level 7–7.5-fold higher than that in the control (Fig. 4). These findings show that *CsERF004* can be up-regulated by SA and ET, suggesting that *CsERF004* may be involved in the SA and ET signalling pathways.

### Subcellular localization analysis

**Fig. 5:**
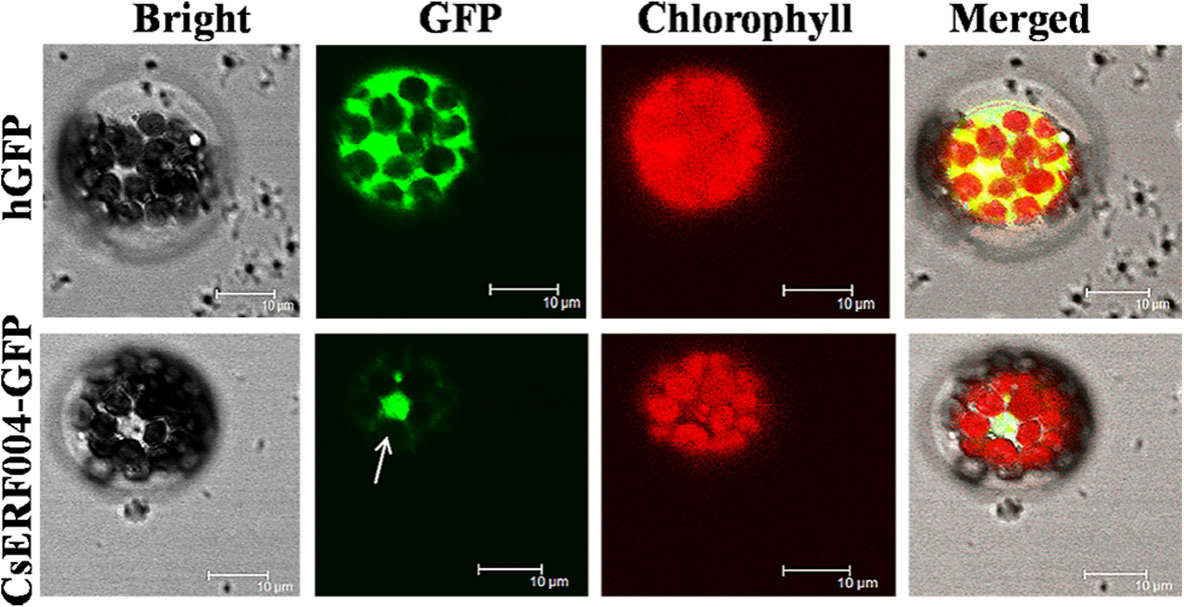
Localization of the CsERF004-GFP fusion protein in *Arabidopsis* protoplasts. The distribution of fluorescent humanized (h)GFP and the CsERF004-GFP fusion protein observed under *white light*, *UV light*, and *red light* are shown. Bars, 10 μm

The CsERF004-GFP fusion expression vector and an empty hGFP vector were individually introduced into *Arabidopsis* protoplasts. The subcellular localization of the CsERF004-GFP fusion protein was observed under a laser scanning confocal microscope. The CsERF004-GFP fusion protein was enriched in the nuclei of *Arabidopsis* cells (Fig. 5), indicating that CsERF004 is a nuclear-localized protein.

### Over-expression of *CsERF004* improves the resistance of cucumber to downy mildew and target spot

**Fig. 6:**
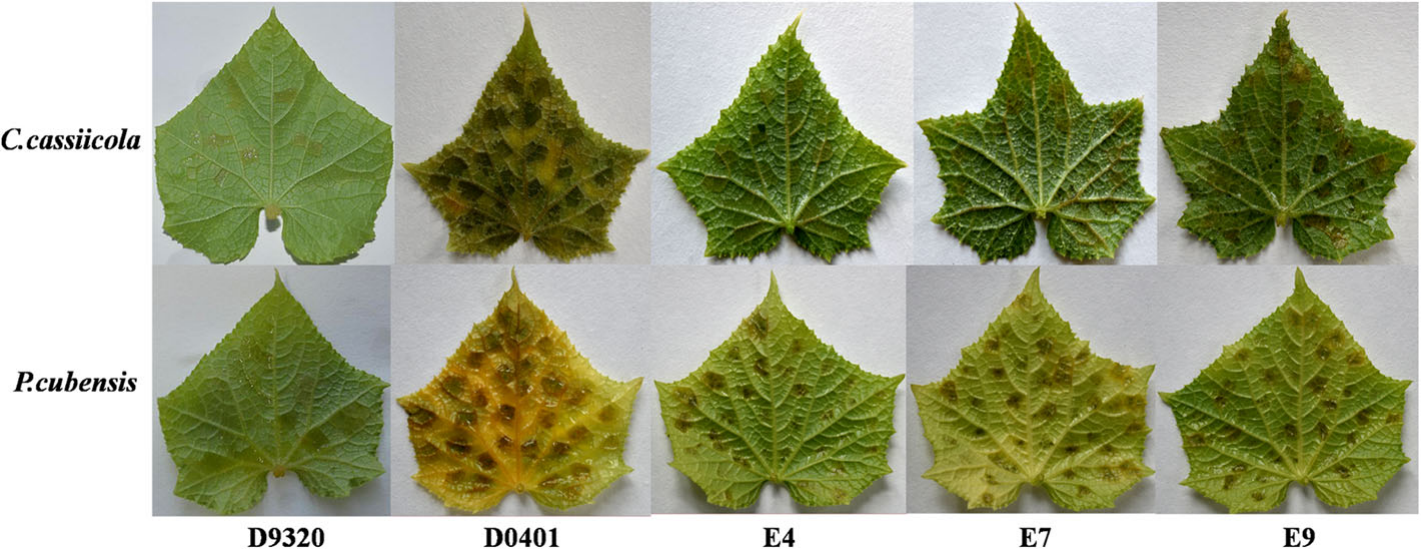
Disease symptoms in over-expressing *CsERF004* T_0_-generation lines E4, E7, and E9. E4, E7, and E9: T_0_-generation represents the three *CsERF004*-over-expressing lines with the highest expression level; WT: control

The over-expression vector PCXSN-*CsERF004* was successfully transferred into the susceptible cultivar D0401 using the cucumber genetic transformation technology. In the T_0_ generation, lines E4, E7, and E9 (with the highest expression levels) were inoculated with *C. cassiicola* and *P. cubensis*. After 7 days, the symptoms of disease in the leaves of the transgenic plants were significantly less severe than those of the susceptible D0401 plants (Fig. 6). T_1_ generation plants over-expressing *CsERF004* were used to analyse disease resistance. After inoculation with *P. cubensis*, the disease index decreased from 90.7 in wild-type plants to 58.7, 62.7, and 60.0 in the E4, E7, and E9 lines, respectively. After inoculation with *C. cassiicola*, the disease index decreased from 85.3 in wild-type plants to 49.3, 52.0 and 56.0 in the E4, E7, and E9 lines, respectively (Table 2). These results show that the over-expression of *CsERF004* can improve the resistance of cucumber to downy mildew and target spot.

**Table 2:** Disease resistance identification of T_1_-generation *CsERF004-*over-expressing plants

Disease name	Disease index			
	E4	E7	E9	WT
Downy mildew (*P. cubensis*)	58.7	62.7	60.0	90.7
Target spot (*C. cassiicola*)	49.3	52.0	56.0	85.3

Disease condition index = Σ (number of disease-level plants × representative levels) × 100/(total number of plants × highest representative value). E4, E7, and E9: T_1_-generation plants in *CsERF004*-over-expressing lines. WT: control

**Fig. 7:**
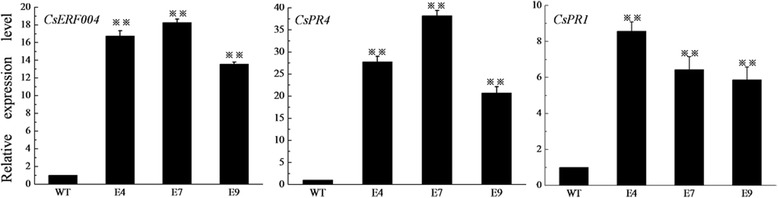
Expression levels of the *CsERF004*, *CsPR1*, and *CsPR4* genes in the T_1_-generation *CsERF004-*over-expressing plants. E4, E7, and E9: T_1_-generation plants; WT: control. There were three biological replicates per treatment and three technical replicates per sample. Student’s t-test was for comparisons between two samples. “**” indicates that the difference between the corresponding column value and the adjacent control column value is significant

The *ERF* genes regulate the expression of *PR* genes, thereby improving plant disease resistance [33–37]. In the present study, the expression levels of the *CsPR1* and *CsPR4* genes were significantly up-regulated after *CsERF004* over-expression. The relative expression of the *CsPR4* gene increased to more than 20-fold that of the control (Fig. 7), and the relative expression of the *CsPR1* gene increased to more than 6-fold that of the control (Fig. 7). Furthermore, we predicted the cis-acting elements of the *CsPR1* and *CsPR4* promoter regions. The *CsPR4* promoter region contained two core CCGAC sequences of DRE/CRT cis-acting elements Additional file 1: Table S1. The *CsPR1* promoter region contained one CCGCC sequence and one CCGTC sequence Additional file 1:Table S1. *LeERF2* has been shown to bind to the CCGCC motif [48]. The over-expression of the *CsERF004* gene may enhance cucumber disease resistance by directly regulating defense genes such as *CsPR1* and *CsPR4*.

### *CsERF004* may require the salicylic acid and ethylene signalling pathways to enhance disease resistance in cucumber

**Fig. 8:**
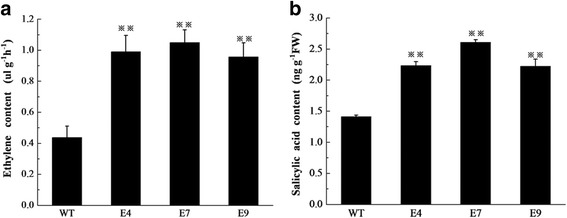
Hormone level analysis of T_1_-generation *CsERF004*-over-expressing plants. a and b indicate ethylene and salicylic acid contents, respectively, in T_1_-generation *CsERF004*-over-expressing plants

SA and ET are important hormones in plant defense responses, and play roles against biotrophic and necrotrophic pathogens, respectively [49]. To further explore the mechanism by which *CsERF004* over-expression enhances the resistance of cucumber to different pathogens, gas chromatography and enzyme-linked immunosorbent assay (ELISA) were performed to determine the ET and SA contents in T_1_ generation plants that over-expressed *CsERF004*. Fig. 8 shows that both the ET and SA contents in *CsERF004*-over-expressing plants were significantly higher than those of wild-type plants. The plants over-expressing *CsERF004* had more than 2-fold more ethylene than wild-type plants (Fig. 8a) and SA levels of approximately 1.5-fold those in the wild-type plants (Fig. 8b). The over-expression of the *CsERF004* gene significantly increased the contents of SA and ET, indicating that *CsERF004* may depend upon the SA and ET signalling pathways to improve cucumber disease resistance.

## Discussion

### *CsERF004* is associated with resistance to downy mildew and target spot in cucumber

Gene expression patterns are often associated with gene function [47]. Under the stress of *P. cubensis*, *C. cassiicola*, or the combination of both pathogens, *CsERF004* was significantly up-regulated in the resistant cultivar in the early stage of pathogen stress, with high and long-lasting expression (Fig. 3a). In the susceptible cultivar, *CsERF004* was significantly up-regulated at 12 h and 120 h after inoculation, although the expression was relatively low and short in duration (Fig. 3b). The expression pattern of *CsERF004* was related to plant disease resistance, which indicated that *CsERF004* plays a role in cucumber resistance to downy mildew and target spot.

### Over-expression of *CsERF004* enhances cucumber resistance to downy mildew and target spot

Previous studies have shown that the up-regulation of ERF enhances plant resistance to single or multiple diseases. Furthermore, the over-expression of the *BrERF11* gene induces the expression of *NtPR-1a/c*, *NtPR3*, and *NtPR-1b* and enhanced the resistance of tobacco to *Ralstonia solanacearum* [50]. The over-expression of *GmERF3* enhanced tobacco resistance to bacterial wilt, brown spot, and *tobacco mosaic virus* [41]. The over-expression of *AaERF1* induced the expression of the *PDF1.2* gene and positively regulated *Artemisia annua* resistance to *Botrytis cinerea* [51]. Chen and Guo introduced the tobacco *OPBP1* gene into rice to enhance its resistance to *Magnaporthe grisea* and *Rhizoctonia solani* [44]. Other studies have shown that ERF negatively regulates plant disease resistance. McGrath et al. [52] and Onate-Sanchez et al. [53] over-expressed *AtERF4* and *AtERF14*, respectively, in *Arabidopsis*, and both reduced the resistance of *Arabidopsis* to Fusarium wilt. The results of the present study showed that the disease symptoms in *CsaERF004*-over-expressing plants were significantly less severe than those of D0401 plants (Fig. 6). Furthermore, the disease index of plants inoculated with downy mildew decreased from 90.7 in wild-type plants to 58.7, 62.7, and 60.0 in the transgenic plants (Table 2). After inoculation with *C. cassiicola*, the disease index decreased from 85.3 in wild-type plants to 49.3, 52.0, and 56.0 in the transgenic plants (Table 2). Therefore, the over-expression of *CsERF004* could improve the resistance of cucumber to downy mildew and target spot. *P. cubensis* is an oomycete, and *C. cassiicola* is a fungus, indicating that *CsERF004* plays an important role in resistance of cucumber to fungal and oomycete infections. Previous studies have shown that the over-expression of *ERFs* not only increases the resistance of plants to fungi, bacteria, and viruses but also improves the resistance of plants to the oomycete family.

### *CsERF004* positively regulates the expression of *CsPR1* and *CsPR4* and enhances cucumber disease resistance

ERF transcription factors are capable of binding to the GCC-box or DRE/CRT cis-acting elements of the gene promoter region to regulate gene expression [31, 32, 48, 54, 55]. Previous studies have shown that the over-expression of *ERFs* in plants enhances plant disease resistance by increasing the expression of *PR* genes. The over-expression of the *GbERF2* transcription factor gene raised the expression levels of *PR-1b*, *PR2* and *PR4* to enhance the resistance of tobacco to *Alternaria longipes* [56]. The over-expression of the *BrERF11* gene up-regulated the expression of *NtPR-1a/c*, *NtPR3*, and *NtPR-1b* and enhanced the resistance of tobacco to *Ralstonia solanacearum* [50]. *PR1* and *PR4* play important roles in plant resistance to pathogens. PR4 has antibacterial activity against *Magnaporthe grisea* and *Fusarium solani* [35, 57]. The over-expression of *PR-1a* in tobacco enhances tobacco resistance to downy mildew and black shank [34]. The results of the present study showed that *CsPR1* and *CsPR4* could be significantly up-regulated after the over-expression of the *CsERF004* gene, which agrees with the findings of previous studies. Compared to that in wild-type plants, the expression of *CsPR4* increased to more than 20-fold, whereas that of *CsPR1* increased to more than 6-fold (Fig. 7). The expression of *CsPR4* was higher than that of *CsPR1*, which may be related to the cis-acting elements in the promoter regions of *CsPR4* and *CsPR1*. The *CsPR4* gene promoter region contains two core CCGAC sequences of DRE/CRT cis-acting elements, and *CsERF004* may regulate expression of *CsPR4* by binding to these CCGAC sequences. The *CsPR1* promoter region has neither a DRE/CRT cis-acting element nor a GCC-box (GCCGCC) cis-acting element and has one CCGCC sequence and one CCGTC sequence. Zhang et al. showed that *LeERF2* binds to the CCGCC box [48]. Wang et al. reported that the third, fourth, and sixth bases of the conserved GCCGCC sequence of the GCC box are essential for ERF protein binding and that other base alterations may affect their binding efficiency [55]. Therefore, the low binding efficiency of CsERF004 and the *CsPR1* promoter may have affected the expression level of *CsPR1*. *CsERF004* may positively regulate the expression of *CsPR1* and *CsPR4*, thereby enhancing disease resistance in cucumber.

### *CsERF004* may require the salicylic acid and ethylene signalling pathways to enhance disease resistance in cucumber

Plants are equipped with different disease resistance signalling pathways that are triggered in response to different infections. The SA disease resistance signalling pathway is mainly involved in resistance to biotrophic pathogens, whereas the ET and JA disease resistance pathways are involved in the resistance to necrotrophic pathogens [49]. *P. cubensis* is a biotrophic pathogen, deriving nutrients from living host tissues [58], and *C. cassiicola* is a necrotrophic pathogen, deriving nutrients from dead or dying cells [59]. There is crosstalk among the SA, ET, and JA signalling pathways, and ERF transcription factors serve as the link among them [38, 39]. *GmERF089* is up-regulated during ET, SA, and JA stress [60]. The expression of *GmERF5* is significantly up-regulated during ET and SA stress and is down-regulated by JA treatment [23]. The results of the present study show that SA and ET, but not JA, could significantly up-regulate the *CsERF004* gene (Fig. 4). The response of the *CsERF004* gene to SA and ET may involve specific signalling pathways in cucumber. The SA and ET contents increased in plants over-expressing *CsERF004*, suggesting that *CsERF004* over-expressing enhances the resistance of cucumber to downy mildew and target spot and that this resistance may be dependent on the SA and ET signalling pathways.

### Implications in plant breeding

Cucumber is a widely cultivated vegetable crop in worldwide. Cucumber downy mildew has occurred in 70 countries [61]. Cucumber target spot has been reported in America, China, Japan and Korea [62]. The two diseases have affected cucumber production. Single-disease-resistant cucumber cultivars have been unable to meet production needs. Therefore, cultivating multi-resistant cucumber cultivars is very important in cucumber production.

This study utilized transcriptome sequencing techniques, screened out the genes related to plant disease resistance. Using transgenic techniques, we analysed gene function and it was confirmed that *CsERF004* is involved in resistance to cucumber downy mildew and target spot. Conventional breeding methods were utilized to cultivate multi-resistant cultivars, which required the identification of multiple resistance genes aiming at different pathogens. The many genes involved may lead to excessively long breeding periods. Identifying the genes related to multi-resistance and utilizing transgenic techniques to support breeding will possible shorten the cultivation period for multi-resistant cultivars.

## Conclusions

In the present study, the *CsERF004* gene, encoding a member of the ERF transcription factor subfamily, was isolated from cucumber and was found to be significantly up-regulated under the stress of *P. cubensis* and *C. cassiicola* infection in resistant varieties and responsive to SA and ET treatments. In terms of the molecular mechanism of cucumber disease resistance, *CsERF004* may promote resistance to *P. cubensis* and *C. cassiicola* in an SA-and-ET-pathway-dependent manner. Based on the analysis of *CsERF004* gene expression patterns and transgenic validation, *CsERF004* is involved in resistance to cucumber downy mildew and target spot. Results could be useful in the development of new resistant cultivars and in understanding the mechanisms of response to pathogens in cucumber.

## Methods

### Plant material

In this study, the homozygous cucumber lines D9320 and D0401 were used as experimental materials. The homozygous cucumber lines D9320 (resistant to downy mildew and target spot) and D0401 (susceptible to downy mildew and target spot) were identified inoculation with *P. cubensis* and *C. cassiicola*, 10 plants per replicate and three replicates tested per treatment [63]. The materials were provided by cucumber research group of Northeast Agricultural University, Harbin, China.

### Pathogen inoculum preparation and inoculation method

*C. cassiicola* isolate of monoconidial cultures (accession number Cc-Tj) was obtained from the Associate Researcher Huizhe Wang (Tianjin Kerun Cucumber Research Institute, Tianjin, China). *P. cubensis* isolate of monosporangial cultures (accession number Pc-hrb6) was obtained from the Professor Yanju Zhang (Northeast Agricultural University, Harbin, China).

Inoculation with *C. cassiicola* [17, 64]: The *C. cassiicola* inoculum was prepared by gently scraping the surface of potato dextrose agar (PDA) medium in sterile water, after which mycelial and conidial suspensions were filtered through four layers of sterile gauze. The conidial suspensions were counted using a haemocytometer and adjusted to 1 × 10^5^ conidia·mL^−1^ using sterile tap water. The first true leaves were inoculated with 30 droplets (approximately 10 μL) of the conidial suspension.

Inoculation with *P. cubensis* [65]: The *P. cubensis* inoculum was prepared by gently scraping the surface of diseased leaves in sterile water, after which sporangia were filtered through four layers of sterile gauze. The sporangial suspensions were counted using a haemocytometer and adjusted to 2.0 × 10^3^ sporangia·mL^−1^ using sterile tap water. The first true leaves were inoculated with 30 droplets (approximately 10 μL) of sporangial suspension.

Simultaneous inoculation with *P. cubensis* and *C. cassiicola*: The first true leaves were inoculated with 15 droplets of the sporangial suspension (2.0 × 10^3^ sporangia·mL^−1^) and 15 droplets of the conidial suspension (1.0 × 10^5^ spores·mL^−1^), each approximately 10 μL in volume.

The culture conditions were as follows: 26 °C/18 °C day and night, and a 12-h/12-h light cycle. Sterile water was used as a control. The three inoculation treatments and the water controls were sampled at 2, 4, 8, 12, 24, 48, 96, and 120 h after inoculation, respectively, frozen in liquid nitrogen, and then stored at −80 °C. Ten plants were inoculated at every time point, and three experimental replicates were performed.

Resistance was assessed by inoculating the T_0_ generation of the E4, E7, and E9 lines with either *C. cassiicola* or *P. cubensis*, and the leaf disease symptoms were photographed (Nikon D5500, Japan) after 7 days. Seeds of the T_1_ generation were obtained via the self-cross of the T_0_ generation lines. A total of 15 individuals from the T_1_ generation (lines E4, E7, and E9) were selected for inoculation, and 15 D0401 plants were inoculated as controls. After inoculation, the disease index was recorded. The severity scale was based on a previous report [17], and the disease index was calculated using the following formula: disease condition index [66] = Σ (number of disease-level plants × representative levels) × 100/(total number of plants × highest representative value)

### Hormone induction treatment

At the two-true-leaf stage of the cucumber cultivar D9320, the leaves were sprayed with 100 μM MeJA (dissolved in was 0.01% ethanol), 1 mM SA (dissolved in sterile water), or 1 mM ET (2 mL 40% EtOH and 1 g NaHCO_3_ dissolved in 200 mL sterile water); sterilized water was used as a control. The ET treatment was conducted in a sealed glass box. Sampling was performed at 6, 12, and 24 h after spraying, and the leaves were then frozen in liquid nitrogen and stored at −80 °C. Ten plants were treated at every time point, and three experimental replicates were performed. Hormone treatment was performed as described elsewhere [23, 67].

### Transcriptome analysis

In this study, the homozygous cucumber lines D9320 and D0401 were used as experimental materials. Samples were taken 48 h after the three inoculation treatments and water control treatments, then frozen in liquid nitrogen and stored at −80 °C. Total RNA was extracted from the cucumber leaves using TRIzol™ (Invitrogen, USA) [48]. RNA samples that met the quality control requirements were sent to Shenzhen Genomics. Then, a cDNA library was obtained by PCR amplification and sequenced on the Illumina HiSeq 2000 platform with 100 cycles of paired-end (2 × 101 bp) sequencing. The raw data (raw reads) were filtered with the FASTQ_Quality_Filter tool from the FASTX-toolkit. The filtered data were used for further analysis. After preprocessing the RNA-Seq data, the reads were mapped to the cucumber genome database (http://cmb.bnu.edu.cn/Cucumis_sativus_v20/) [68]. Differentially expressed genes were identified based on a *p* value ≤0.01 and | log2 ratio ≥ 1 |.

### Gene cloning and bioinformatics analysis

The full-length CDS of the cucumber gene ID Csa7M432080.1 (*CsERF004*) was searched in the cucumber genome database, and specific primers for cloning the full-length CDS were designed using Primer Premier 5.0 (Table 3). PCR was used to obtain the full-length CDS of *CsERF004*. The PCR conditions were as follows: denaturation at 94 °C for 30 min, annealing at 50 °C for 30 s, extension at 72 °C for 10 min for a total of 30 cycles; and a final extension at 72 °C for 10 min. The amplified products were stored at 4 °C.

**Table 3:** Primer sequences

Primer name	Sequence (5′-3′)	Used for
CsERF004-F	ATGGCTCGTCCACAACAACG	Cloning
CsERF004-R	TTATGTAATAATTTCGAATGATCCGAG	Cloning
qCsERF004-F	CAACACGTCCACAAACAACA	qRT-PCR
qCsERF004-R	TGGAATAGACCCACGTTTCA	qRT-PCR
qCsPR1-F	TGGTGCACTCCAATGGCTCT	qRT-PCR
qCsPR1-R	GGGTGTAATGGCGACACTCG	qRT-PCR
qCsPR4-F	ACTGCTTTCTGTGGCCCAGTT	qRT-PCR
qCsPR4-R	ACCCTCCGTTGGAGCATTGA	qRT-PCR
qCsEF1a-F	CCAAGGCAAGGTACGATGAAA	qRT-PCR
qCsEF1a-R	AGAGATGGGAACGAAGGGGAT	qRT-PCR
qCsEF1a-R	AGAGATGGGAACGAAGGGGAT	qRT-PCR
GFP-CsERF004-F	GGATCCATGGCTCGTCCACAACAACG	Subcellular localization
GFP-CsERF004-R	GGACCCGGGTTGTAATAATTTCGAATGATCC	Subcellular localization

The analysis of the conserved domains in the CsERF004 protein was performed using the NCBI CDD database for conserved protein domains (https://www.ncbi.nlm.nih.gov/Structure/cdd/wrpsb.cgi). A phylogenetic tree was constructed using MAGE5.10 software. The sequence alignment of the conserved domains was performed using DNAMAN software. All sequences data were obtained from the National Center for Biotechnology Information (NCBI) Additional file 2:Table S2.

### Promoter sequence analysis

BLAST sequence alignment was performed in the cucumber genome database (http://www.icugi.org/) to obtain the complete sequence of the gene; the sequence 2000 bp upstream of the transcription initiation region was considered the promoter region. The online tool PlantCARE (http://bioinformatics.psb.ugent.be/webtools/plantcare/html/) and the PLACE database (http://www.dna.affrc.go.jp/htdocs/PLACE/) were used to perform promoter sequence analysis.

### qRT-PCR analysis

Samples were taken 2, 4, 8, 12, 24, 48, 96, and 120 h after inoculation or water control treatment. Samples from the three hormone induction treatments and water controls were taken 6, 12, and 24 h after spraying. Total RNA was extracted from cucumber leaves using the TRIzol (Invitrogen, USA) method [69]. The first-strand cDNA was reverse-transcribed using a Toyobo reverse transcription kit ReverTra Ace qPCR RT-Kit (ToYoBo, Japan). The quality and concentration of the cDNA were determined using a SMA3000 UV spectrophotometer (Beijing, China). After dilution, the cDNA was stored at −20 °C until use.

The qRT-PCR primers were designed using the online tool GenScript Real-time PCR (TaqMan) Primer Design (https://www.genscript.com/ssl-bin/app/primer) (Table 3). qRT-PCR was performed using SYBR® Green Master Mix (ToYoBo, Japan) in an iQ5 (Bio-Rad) thermocycler. *CsEF1α* (XM_004138916) [70] was used as the housekeeping gene. Three biological replicates per treatment and three technical replicates per sample were analysed. The relative gene expression was calculated using the 2^−ΔΔCT^ relative quantitative analysis method [71], and variance and significance were analysed with DPS 7.05 data processing system software. Significant differences between the treatment and the control were determined using Student’s t- test.

### Subcellular localization

A *CsERF004* and *GFP* gene fusion vector was constructed, primers with enzyme digestion sites for *Bam*HI and *Sma*I were designed (Table 3), and the *CsERF004* open reading frame was amplified without its stop codon. The fusion expression vector pSASY-T3-*CsERF004* and the transient expression vector pGII-eGFP were digested with *Bam*HI and *Sma*I, and the product recovered from the gel was ligated to obtain the fusion expression vector 35S–*CsERF004*-eGFP. The *A. thaliana* (Columbia ecotype) were obtained from the European Arabidopsis Stock Centre (NASC, Nottingham, UK). The *A. thaliana* protoplasts were extracted and transformed as described elsewhere [72]. Subcellular localization was observed using confocal laser scanning microscopy (Leica, Germany).

### Genetic transformation of the susceptible cultivar D0401

The full-length CDS of the *CsERF004* gene obtained using PCR was ligated into the PCXSN-1250 vector, which had been digested with *Xcm*I [73], to yield the over-expression vector PCXSN*-CsERF004*. PCXSN*-CsERF004* and PCXSN-1250 plasmids were transferred into *Agrobacterium tumefaciens* LBA4404 (BioVector NTCC Inc., Beijing, China) using the freeze-thaw method [74].

The susceptible cultivar D0401 was transformed with the over-expression vector PCXSN*-CsERF004* using the cucumber genetic transformation technology [75] described by Zhang et al. and grown on a concentration of 1 mg/L glufosinate.

### Expression analysis and physiological index determination of *CsERF004*-over-expressioning plants

The expression of *CsPR1* and *CsPR4* was analysed by qRT-PCR in *CsERF004*-over-expressing plants. The ET content was determined by gas chromatography [76]. SA extraction was performed as described elsewhere [77], and a plant SA ELISA kit (Shanghai, China enzyme biotechnology Co., Ltd.) was used to determine the SA content.

## Additional files

Additional file 1: Table S1. Locations and sequences of cis-elements in the promoter regions of the *CsPR1* and *CsPR4* genes. (docx 19.4 KB)

Additional file 2: Table S2. All sequences data in Fig. 2. (docx 16.7 KB)

## Abbreviations

cDNA: Complementrary DNA
ET: Ethylene
MeJA: Methyl jasmonate
PCR: Polymerase chain reaction
PDA: Potato dextrose agar medium
PR1: Pathogenesis-related protein 1-like
PR4: Pathogenesis-related protein 4A
qRT-PCR: Real-time quantitative reverse transcription-polymerase chain reaction
RNA: Ribonuncleic acid
SA: Salicylic acid
