# Supplementary material for: Expression and functional analysis of the transcription factor-encoding Gene CsERF004 in cucumber during Pseudoperonospora cubensis and Corynespora cassiicola infection
Source: BMC Plant Biol. 2017 Jun 5;17:96. doi: 10.1186/s12870-017-1049-8 (PMC5460474; doi:10.1186/s12870-017-1049-8)
Supplement: Supplementary file 1 — Locations and sequences of cis-elements in the promoter regions of the CsPR1 and CsPR4 genes. (docx 19.4 KB) [file 12870_2017_1049_MOESM1_ESM.docx]

**Table S1.** Location and sequence of cis- elements in *CsPR1* and *CsPR4* promoter.

| **Gene ID** | **Gene name** | **Sequence** | Local |
| --- | --- | --- | --- |
| Csa7M070250.1 | *CsPR1* | CCGTC | −671–−675 |
| Csa7M070250.1 | *CsPR1* | CCGCC | −903–−907 |
| Csa2M010380.1 | *CsPR4* | CCGAC | −1219–−1223 |
| Csa2M010380.1 | *CsPR4* | CCGAC | −1303–−1307 |
