# Supplementary material for: Expression and functional analysis of the transcription factor-encoding Gene CsERF004 in cucumber during Pseudoperonospora cubensis and Corynespora cassiicola infection
Source: BMC Plant Biol. 2017 Jun 5;17:96. doi: 10.1186/s12870-017-1049-8 (PMC5460474; doi:10.1186/s12870-017-1049-8)
Supplement: Supplementary file 2 — All sequences data in Fig. 2. (docx 16.7 KB) [file 12870_2017_1049_MOESM2_ESM.docx]

**Table S2.** All sequences data in Figure 2.

| **GeneBank**  **accession number** | **Protein name** | **Sequence** |
| --- | --- | --- |
| AAC49777.1 | AtRAP2.11 | KNMEHQTTPKQKTKEKSKGNKTKFVGVRQRPSGKWVAEIKDTTQKIRMWLGTFETAEEAARAYDEAACLLRGSNTRTNFANHFPNNSQLSLKIRNLLHQKQSMKQQQQQQHKPVSSLTDCNINYISTATSLTTTTTTTTTTAIPLNNVYRPDSSVIGQPETEGLQLPYSWPLVSGFNHQIPLAQAGGETHGHLNDHYSTDQHLGLAEIERQISASLYAMNGANSYYDNMNAEYAIFDPTDPIWDLPSLSQLFCPT |
| JAU24088.1 | NcRAP2-11 | CLCTDSYKKMEHQTTQKQKTKEKSKGNKTKFVGVRQRPSGKWVAEIKDTTQKIRMWLGTFETAEEAARAYDEAACLLRGSNTRTNFANHFPNNSQLSLKIRNLLHQKQSMKQQQQQQQQHKPVSSFSECSINYTSTATSLTATTTMPVNNVYRPDSSVIRQAETDGVQLPYSWPHVSGFNHQVPLDQGGEEANGHLNDQNPTDQHLGLAEIERQISASLYAMNGANSYYDNMNAEYAIFDPTDPIWDLPSLSQLFCPT |
| XP_010493057.1 | CaRAP2-11 | MERQQPQKQKTKEKSKGNKTKFVGVRQRPSGKWVAEIKDTTQKIRMWLGTFETAEEAARAYDEAACLLRGSNTRTNFANHFPNNSQLSLKIRNLLHQKQSMKQQQQQQHKPVSSFAECNINYTSAAATSITTTTTTTTAIPLNDVYRPDSSVIGLPETTGHQHPYSWPLVSGFNHLVPMAQGGEETHGHLNDHNSTDQHLGLAEIERQISASLYAMNGPNSYYDSMNAEYAIFDPTDPIWDLPSLSQLFCPT |
| XP_013667343.1 | BnRAP2-11-like | MENKTTQKQKTKEKSKSNNKTKFVGVRQRPSGKWVAEIKDTTQKIRMWLGTFETAEEAARAYDEAACLLRGSNTRTNFANHFPNNSQLSLKIRNLLHQKQTMKQQQQKPKQNKPVSSFAECSINYTSNATTLTSTTTTTTVLPLNNVYRPDSSVISHPEADSVQLPYTWPLVSGFNHHVPLAQGGEEPHGHLNNQNSTDQHLGLADIERQISASLYAMNGANGYYDNMNTEYAIFDPTDPIWDLPSLSQLFCPT |
| NP_197901.1 | AtESE3 | MARPQQRFRGVRQRHWGSWVSEIRHPLLKTRIWLGTFETAEDAARAYDEAARLMCGPRARTNFPYNPNAIPTSSSKLLSATLTAKLHKCYMASLQMTKQTQTQTQTQTARSQSADSDGVTANESHLNRGVTETTEIKWEDGNANMQQNFRPLEEDHIEQMIEELLHYGSIELCSVLPTQTL |
| NP_567530.4 | AtERF1 | MSMTADSQSDYAFLESIRRHLLGESEPILSESTASSVTQSCVTGQSIKPVYGRNPSFSKLYPCFTESWGDLPLKENDSEDMLVYGILNDAFHGGWEPSSSSSDEDRSSFPSVKIETPESFAAVDSVPVKKEKTSPVSAAVTAAKGKHYRGVRQRPWGKFAAEIRDPAKNGARVWLGTFETAEDAALAYDRAAFRMRGSRALLNFPLRVNSGEPDPVRIKSKRSSFSSSNENGAPKKRRTVAAGGGMDKGLTVKCEVVEVARGDRLLVL |
| JAU16327.1 | NcERF1a | MAAESDYAFLESIRRHLLGESEAPRLSSESMASSGEQSRTTGHTLKPVYGRNPSFSKLYPCFTESWGDLPLKENDSEDMLVYGILNDAFHGGWEPSSSSSDEDRSSFPAVKIETPESFKAVDSAPVKKTSPVPAAAKGKHYRGVRQRPWGKFAAEIRDPAKNGARVWLGTFETAEDAALAYDRAAFRMRGSRALLNFPLRVNSGEPDPVRIKSKRASASDNGASKRRRTVASVNAAGQGTDNGLKVKCEVVEMTRGDQLLVL |
| CAB45963.1 | AtEREBP-2 | GQSIKPVYGRNPSFSKLYPCFTESWGDLPLKENDSEDMLVYGILNDAFHGGWEPSSSSSDEDRSSFPSVKIETPESFAAVDSVPVKKEKTSPVSAAVTAAKGKHYRGVRQRPWGKFAAEIRDPAKNGARVWLGTFETAEDAALAYDRAAFRMRGSRALLNFPLRVNSGEPDPVRIKSKRSSFSSSNENGAPKKRRTVAAGGGMDKGLTVKCEVVEVARGDRLLVL |
| NP_199533.1 | AtERF2 | MYGQCNIESDYALLESITRHLLGGGGENELRLNESTPSSCFTESWGGLPLKENDSEDMLVYGLLKDAFHFDTSSSDLSCLFDFPAVKVEPTENFTAMEEKPKKAIPVTETAVKAKHYRGVRQRPWGKFAAEIRDPAKNGARVWLGTFETAEDAALAYDIAAFRMRGSRALLNFPLRVNSGEPDPVRITSKRSSSSSSSSSSSTSSSENGKLKRRRKAENLTSEVVQVKCEVGDETRVDELLVS |
| NP_001275437.1 | Pti4 | MDQQLPPINFPVDFPVYRRNSSFSRLIPCLTETWGDLPLKVDDSEDMVIYGLLKDALSVGWSPFSFTAGEVKSEPREEIESAPEFVPSPVETTAAPAAETPKGRHYRGVRQRPWGKFAAEIRDPAKNGARVWLGTYETAEEAAIAYDKAAYRMRGSKAHLNFPHRIGLNEPEPVRVTAKRRASPEPVSSSENGSMKRRRKAVRKCDGGVESRSSAIQIGCQIEQLTGVHQLLVI |
| NP_850582.1 | AtRAP2.2 | MCGGAIISDFIPPPRSLRVTNEFIWPDLKNKVKASKKRSNKRSDFFDLDDDFEADFQGFKDDSAFDCEDDDDVFVNVKPFVFTATTKPVASAFVSTVGSAYAKKTVESAEQAEKSSKRKRKNQYRGIRQRPWGKWAAEIRDPRKGSREWLGTFDTAEEAARAYDAAARRIRGTKAKVNFPEEKNPSVVSQKRPSAKTNNLQKSVAKPNKSVTLVQQPTHLSQQYCNNSFDNSFGDMSFMEEKPQMYNNQFGLTNSFDAGGNNGYQYFSSDQGSNSFDCSEFGWSDHGPKTPEISSMLVNNNEASFVEETNAAKKLKPNSDESDDLMAYLDNALWDTPLEVEAMLGADAGAVTQEEENPVELWSLDEINFMLEGDF |
| NP_175794.1 | AtRAP2.12 | MCGGAIISDFIPPPRSRRVTSEFIWPDLKKNLKGSKKSSKNRSNFFDFDAEFEADFQGFKDDSSIDCDDDFDVGDVFADVKPFVFTSTPKPAVSAAAEGSVFGKKVTGLDGDAEKSANRKRKNQYRGIRQRPWGKWAAEIRDPREGARIWLGTFKTAEEAARAYDAAARRIRGSKAKVNFPEENMKANSQKRSVKANLQKPVAKPNPNPSPALVQNSNISFENMCFMEEKHQVSNNNNNQFGMTNSVDAGCNGYQYFSSDQGSNSFDCSEFGWSDQAPITPDISSAVINNNNSALFFEEANPAKKLKSMDFETPYNNTEWDASLDFLNEDAVTTQDNGANPMDLWSIDEIHSMIGGVF |
| NP_001238300.1 | GmAP2-EREBP | MCGGAIISDFIPAAAIAGSRRLTADYLWPDLKKRKSDLDVDFEADFRDFKDDSDIDDDDDDHQVKPFAFAASSRLSTAAKSVAFQGRAEISANRKRKNQYRGIRQRPWGKWAAEIRDPRKGVRVWLGTFNTAEEAARAYDAEARRIRGKKAKVNFPEAPGTSSVKRSKVNPQENLKTVQPNLGHKFSAGNNHMDLVEQKPLVSQYANMASFPGSGNGLRSLPSSDDATLYFSSDQGSNSFDYAPEISSMLSAPLDCESHFVQNANQQQPNSQNVVSIEDDSAKTLSEELVDIESELKFFQMPYLEGSWGDTSLESLLSGDTTQDGGNLMNLWCFDDIPSMAGGVF |
| NP_001238595.1 | GmERF4 | MAPRDHKTSNAKANGNGNSGVKEVHFRGVRKRPWGRYAAEIRDPGKKSRVWLGTFDTAEEAARAYDAAAREFRGPKAKTNFPLPLENVKNSSPSQSSTVESSSRDRDVAADSSPLDLNLAPAAAASARFPFQHQFPVFTGAVPAANQVLYFDAVLRAGMAGPRGFAFGYNHHPVAASEFHATTSDSDSSSVIDLNHNEGEVKGNGSRIFDLDLNHPPPHEIA |
| NP_188139.1 | AtERF4 | MAKMGLKPDPATTNQTHNNAKEIRYRGVRKRPWGRYAAEIRDPGKKTRVWLGTFDTAEEAARAYDTAARDFRGAKAKTNFPTFLELSDQKVPTGFARSPSQSSTLDCASPPTLVVPSATAGNVPPQLELSLGGGGGGSCYQIPMSRPVYFLDLMGIGNVGRGQPPPVTSAFRSPVVHVATKMACGAQSDSDSSSVVDFEGGMEKRSQLLDLDLNLPPPSEQA |
| AAC49771.1 | AtRAP2.5 | MAKMGLKPDPATTNQTHNNAKEIRYRGVRKRPWGRYAAEIRDPGKKTRVWLGTFDTAEEAARAYDTAARDFRGAKAKTNFPTFLELSDQKVPTGFARSPSQSSTLDCASPPTLVVPSATAGNVPPQLELSLGGGGGGSCYQIPMSRPVYFLDLMGIGNVGRGQPPPVTSAFRSPVVHVATKMACGAQSDSDSSSVVDFEGGMEKRSQTVRSRS |
| JAU43921.1 | NcERF4 | TEAFGPSIITPLSLTSLSLYLRFLVMVKMGLKPDPATPHPTQNNAKEIRYRGVRKRPWGRYAAEIRDPGKKTRVWLGTFDTAEEAARAYDAAARDFRGAKAKTNFPTFLELSEKVPVGGGGFARSPSQSSTLDCASPPTAVAPVTTNNIPPQLELSLGGGACYQIPMARPVYFLDLMGIGNGGRGQPPVSSAFRSSVVHVATKTACAQSDSDSSSVVDFEGGMEKRSQPLDLDLNLPPPSEQA |
| XP_011659507.1  (Csa7M432080.1 is ID in the cucumber genome database) | CsERF004 | MARPQQRYRGVRQRHWGSWVSEIRHPLLKTRIWLGTFETAEDAARAYDEAARLMCGPKARTNFPYNPNDQQSSSSFSSSSKLLSAALIEKLHKCHLASLQIAKQHVHKQHAGFEPSYLAYSGSPPPIITGATTSQWASDETWVYSNKGDQMEMNNNNNNYNNNIHHQQCQLEPLEDDHIEQMIQELLDLGSFEIIT |
